# Parental Experiences of Adolescent Cancer-Related Pain: A Qualitative Study

**DOI:** 10.1093/jpepsy/jsac047

**Published:** 2022-05-20

**Authors:** Charlotte Clews, Cara Davis, Maria Loades, Abbie Jordan

**Affiliations:** Department of Psychology, University of Bath, UK; Department of Psychology, University of Bath, UK; Department of Psychology, University of Bath, UK; Bristol Medical School, University of Bristol, UK; Department of Psychology, University of Bath, UK; Centre for Pain Research, University of Bath, UK

**Keywords:** adolescent, cancer, cancer-related pain, pain management, parent experience, qualitative

## Abstract

**Objective:**

Despite advancing medical treatments, pain remains a significant outcome of adolescent cancer, as both a problematic and distressing symptom. With adolescents spending substantial periods of time at home during cancer treatment, parents perceive themselves as central to the experience and management of adolescents’ pain. The present study aimed to explore parental experiences of adolescent cancer-related pain during, and recently after, completing cancer treatment.

**Methods:**

We interviewed 21 parents of adolescents (aged 12–18 years) with cancer, recruited through a hospital in South West England. Interviews were analyzed using reflexive inductive thematic analysis.

**Results:**

Two themes were generated. The first theme, “Parental perceptions of being at the heart of pain management,” focused on the role of parents in adolescents’ pain journeys, and the vast knowledge they gained. The second, “Adapting and readjusting expectations,” captured parents’ journeys in learning to adjust their lives according to adolescents’ pain and difficulties they faced throughout this process.

**Conclusions:**

Findings highlighted parents’ crucial role throughout adolescents’ pain experiences; learning how to manage adolescents’ pain, and supporting them with the detrimental impact on their lives. The findings emphasize the importance of a multidisciplinary approach to supporting families to manage pain. They also indicate a need for targeted research studies investigating parental experiences of adolescent cancer-related pain. This will help professionals understand how best to support parents and adolescents throughout the cancer journey and ultimately improve the physical and psychological outcomes of young people in the longer term.

## Introduction

Pain is a common experience in adolescence, for example in relation to chronic back pain, stomach pain, headaches, and cancer (Gobina et al., 2019; Linder & Hooke, 2019). Studies describe pain prevalence amongst adolescents with cancer to be 28–62% (Linder & Hooke, 2019), with adolescents reporting pain as causing moderate–great distress throughout the cancer journey (Van Cleve et al., 2012). Pain is one of the most common initial symptoms leading to cancer diagnoses, and thereafter frequently perceived as a consequence of the cancer, treatments, and/or procedures (Duffy et al., 2019). Pain is considered by many adolescents as being an inevitable part of the cancer journey (Woodgate & Degner, 2003). Extending the experience of pain in the context of adolescent cancer, parents report pain to be the most problematic and distressing aspect of cancer treatment for adolescents (Duffy et al., 2019). At a time in adolescents’ lives where independence normatively increases, cancer-related pain can mean they deviate from this course of development, and many become more dependent on their parents (Drew et al., 2019). Pain can be especially difficult to manage in adolescents with cancer. In part, this may be due to unique developmental concerns within this group that conversations about pain will elicit unwanted reactions, such as restrictions of social and other age-related activities (Ameringer, 2010). Many adolescents with cancer fear their pain will be ignored or not taken seriously (Uhl et al., 2020), meaning they are likely to under-report, and in some cases even deny, pain (Ameringer, 2010; Duffy et al., 2019).

The context of adolescent cancer extends beyond the adolescent, with parents acting as the first point of contact if and when adolescents seek further explanation for any new or ambiguous pains that they experience post cancer diagnosis and treatment (Tutelman et al., 2019). With treatment advances resulting in increased outpatient care for adolescents with cancer, the responsibility of care is shifted from healthcare professionals onto parents (Parker et al., 2021). Common responsibilities of parents involve recognizing and assessing adolescent’s pain, deciding which pain relief to administer, and supporting implementation of pain management strategies (Parker et al., 2020; Uhl et al., 2020). Despite the perceived benefits of home-based management of adolescent cancer for families, adolescents and parents have access to fewer cancer pain-management strategies compared with hospital management (Jibb et al., 2014). Whilst research identifies a lack of evidence-based strategies for parental management of adolescent pain in a home setting (Twycross et al., 2015), parents of adolescents with cancer report using multiple techniques to manage pain symptoms (Linder et al., 2017). Interestingly, parents often under-administer pain medications (Fortier et al., 2014), with evidence suggesting they do not consider their adolescents’ pain to be sufficiently severe to require pain relief, or that parents think adolescents are exaggerating their pain levels or seeking attention (Twycross et al., 2015). Many parents of children and adolescents living with cancer report their experience of caring for their child to be stressful, and describe a sense of perceived helplessness and inadequacy in comforting their child (Rodriguez et al., 2012; Twycross et al., 2015). Such emotional experiences may leave parents of adolescents with cancer vulnerable to psychological consequences, including post-traumatic stress disorder, anxiety, and depression (Alberts et al., 2018). These consequences are often associated with a higher use of disengagement coping strategies, a desire for their adolescent’s condition to improve, as well as self-criticism and blaming themselves for their adolescent’s illness (Trask et al., 2003). It is therefore essential that parents’ experiences of managing their adolescent’s pain are clearly understood to enable parents to support adolescents using evidence-based strategies and to promote positive psychological outcomes.

Whilst an abundance of studies exist in the area of adolescent cancer-related pain, the majority have focused on the prevalence of cancer-related pain and the types of management strategies parents and adolescents use. Few studies have explored parental perceptions of adolescent’s cancer-related pain and parental experiences of managing adolescent’s cancer-related pain. Typically, existing studies have adopted a nomothetic approach, resulting in an inability to understand the complexity of parental perceptions and experiences of adolescent pain management in the context of cancer (Parker et al., 2020). To address this knowledge gap, the present study aimed to generate a detailed idiographic understanding of parents’ experiences with adolescent cancer-related pain during, or having recently completed, active cancer treatment, and how parents support their adolescents to manage their pain symptoms.

## Methods

The present study formed the pain element of a wider qualitative research project exploring parental experiences of adolescent cancer-related fatigue (Loades et al., 2020) and distress (Sharma et al., 2021). Ethical approval was granted by the NHS Research Ethics Committee (8/SW/0222), and university departmental Research Ethics Committee (18-303).

### Participants

Individuals were eligible to take part in the study if they were parents or carers of adolescents (a) aged 12–18 years, with a diagnosis of cancer. The age range selected reflects the average age of pubertal onset (World Health Organization, 2014), and typically the start of secondary school in England, which occurs at 11/12 years; (b) receiving care at, or known to, the recruitment site (a hospital in South West England); (c) receiving primary active treatment (or maintenance treatment for acute lymphoblastic leukemia), or up to 1-year post-treatment, when oncology services were most involved, and could support parents and adolescents; and (d) whose distress levels were deemed by healthcare professionals to be sufficiently low that participation would not exacerbate their distress. Participants were recruited between March and July 2019, through the pediatric oncology and hematology team, and teenage and young adult service within the hospital. Purposive sampling was chosen to recruit participants, a sampling approach which is commonly adopted when researching health and sensitive topics (Campbell et al., 2020). Healthcare professionals identified potentially eligible parents, and provided them with information about the study and contact details of the researchers. The original study aimed to recruit 15–25 participants, based on previous qualitative studies investigating similar topic areas, including parents’ perceptions of cancer pain and other cancer-related symptoms (Bj**ö**rk et al., 2005; Parker et al., 2021).

Of the parents approached, 26 expressed an interest in participation; 5 parents were unable to participate due to: (a) being ineligible (*N*** **=** **1); (b) being non-contactable (*N*** **=** **1); and (c) having other commitments (*N*** **=** **3). The final sample comprised 21 parents aged 42–57 years of 17 adolescents aged 12–18 years (15 mothers and 6 fathers). Participants were interviewed face-to-face (*N*** **=** **15; 8 at home, 1 at their workplace, 6 at the recruitment hospital), or over the telephone (*N*** **=** **6). Treatment duration of adolescents on current active treatment ranged from 2 to 24 months (*M*** **=** **7.36, *SD*** **=** **6.04). Parents reported adolescents who had completed active treatment to have received treatment lasting between 4 and 27 months (*M*** **=** **10.83, *SD*** **=** **8.28). Parents were asked to rate the extent to which pain was perceived to be problematic for their adolescent using a numerical 1–10 rating scale, with a score of 10 indicating the most problematic pain (*M*** **=** **6.93, *SD*** **=** **2.55). Parent and adolescent characteristics of the final sample are presented in Table I. Data pertaining to participants’ race and ethnicity were not collected and are unfortunately not obtainable retrospectively.

**Table I.** Parent and Adolescent Demographics and Clinical Characteristics

	Parent interviewed	Adolescent age (years)	Adolescent gender	Adolescent’s cancer type	Pain rating	On/off treatment
P1	Mother	16	Female	Acute lymphoblastic leukemia	10	On
P2	Mother	17	Female	Osteoblastic sarcoma	10	Off
P3	Father	15	Male	Acute myeloid leukemia	3	Off
P4	Mother	13	Female	Acute myeloid leukemia^a^	5	On
P5	Mother	18	Female	Ewing sarcoma	8	Off
P6	Father	18	Female	Thyroid cancer	0	On
P7	Mother				0	
P8	Mother	16	Male	Brain/germ cell tumor	4–10	On
P9	Father	18	Female	Germ cell tumor	7	Off
P10	Mother				8	
P11	Mother	15	Female	Acute lyphoblastic leukemia	5	Off
P12	Mother	15	Female	Osteosarcoma	10	Off
P13	Mother	17	Female	Hodgkin’s lymphoma	1	On
P14	Father	16	Female	Hodgkin’s lymphoma	5/6	On
P15	Mother	14	Female	Osteosarcoma	8	On
P16	Father				2/3	
P17	Mother	18	Female	Acute lymphoblastic leukemia	5	On
P18	Mother	18	Male	Germ cell cancer	8/9	N/A
P19	Mother	12	Male	Brain tumor—optic glioma	9	On
P20	Father				7	
P21	Mother	12	Female	Multifocal cutaneous anaplastic large cell lymphoma	6–10	On

*Note.* Participants highlighted in gray indicate parental dyads.

a Adolescent was previously diagnosed with leukemia and had relapsed.

### Procedure

Participants were interviewed individually by one of two female Masters in Health Psychology students, supervised by a clinical psychologist (L.B., see Acknowledgments). The interviewers had completed Masters modules in qualitative interviewing, and practiced using the topic guide before the interviews to increase familiarization. The topic guide and regular supervisions during the interview phase ensured consistency and rigor between interviewers. Prior to interview, participants were given information about the study and provided informed consent. Demographic information was obtained, including parent and adolescent age, gender, adolescent cancer type, and treatment (Table I). Semi-structured interview schedules comprised five open-ended questions, plus prompts to explore topics in greater detail, examining parents’ perceptions of adolescent’s fatigue, distress, and pain (see Table II for the pain questions). Questions were designed in collaboration with adolescents with lived experience of cancer treatment, and clinicians working in cancer services. This ensured relevance of the research design, and that the proposed questions were ethically acceptable and appropriate to this particular population (Bagley et al., 2016). Interviews were digitally recorded, transcribed verbatim, and checked for accuracy by members of the research team. Interview duration ranged from 18 to 80 min (*M*** **=** **39, *SD*** **=** **15). All identifiable participant information was removed at transcription, and participants were assigned a number.

**Table II.** Topic Guide (Pain Questions Only)

*Pain*
Some parents have told us that pain is a problem for their child.
Can you tell me your son’s/daughter’s experience of pain?
How well do you feel you understand your son’s/daughter’s pain?
What have you found to be the most helpful ways of managing your child’s pain?
Can you tell me about any resources or support that you have found beneficial or would have liked to have been offered?
What advice would you give to other parents of children with cancer about managing their children’s pain?

### Data Analysis

Inductive reflexive thematic analysis (Braun & Clarke, 2006, 2019) was selected to analyze the qualitative interview-generated data, due to its focus on investigating parents’ realities and the meaning of their experiences of having an adolescent who was receiving or had received cancer treatment. Using an inductive approach meant codes and themes were derived from the data as a whole (Braun & Clarke, 2012), an approach applied in other research in similar areas (e.g., Sharma et al., 2021), as opposed to using pre-existing theory. The analysis was informed by a critical realist perspective, which distinguishes between the participant’s “real” and “observable” world (Fletcher, 2017). Consequently, we aimed to understand parents’ reality of their perceptions and experiences of their adolescent’s pain, by understanding how parents interpreted observable events in their daily lives, and how these developed throughout their adolescent’s cancer journey.

Analysis was conducted by CC using NVivo (Version 12; QSR International), following the six stages of inductive thematic analysis described by Braun and Clarke (2006, 2019). The first stage involved the researcher repeatedly reading transcripts to familiarize themselves with the data. Initial notes of impressions and items relevant to the research question were made. In the second stage, coding was conducted using NVivo and was a complete process, meaning everything relevant to parents’ experiences of their adolescent’s cancer-related pain was assigned a code (Braun & Clarke, 2012). Semantic and latent meaning were identified from the data, enabling the analysis to capture the content and meaning of parents’ experiences (Braun & Clarke, 2019). The third stage involved identifying patterns across the data. Codes and corresponding quotations with shared meanings were grouped into potential themes and subthemes, allowing recurring patterns across the dataset to be captured meaningfully in relation to the research question (Braun & Clarke, 2012). The fourth stage involved regularly reviewing codes, and making frequent changes to the organization of the themes and subthemes to ensure the analysis related meaningfully to the research aims, and so the themes did not overlap. C.C. reviewed and refined themes with the wider supervisory team (C.D. and A.J.). In the fifth stage, two themes were generated, named and defined in a way that best captured the overall meaning of the data and our interpretation. The final stage involved writing the report. Quotations best representing each theme were selected, and interpreted in relation to the aims of the study.

Quality issues with regard to qualitative research were addressed in numerous ways. The authors first acknowledged the depth of their experiences and biases that they might have brought to the interpretation of the data, including experience as a female Masters in Health Psychology student on a placement within pediatric and adolescent oncology (C.C.), a female clinical psychologist with over 10 years of experience working within pediatric health (C.D.), and as a female health psychologist with 20 years of experience of working in the field of adolescent pain (A.J.). Additionally, the research question, aims, and the steps of reflexive thematic analysis were clearly outlined and explained to improve research clarity (Kitto et al., 2008). Transparency of the analysis process was maintained by C.C. keeping a reflexive journal, critically reflecting on their role as a researcher and how personal experience of cancer may have shaped the findings produced (Braun & Clarke, 2012). Steps were taken to enhance the credibility of the findings by ensuring quotations from the data were provided from across the sample so that participants’ diverse experiences were represented (Kitto et al., 2008).

## Findings

Two themes were generated: (a) “Parental perceptions of being at the heart of pain management,” highlighting parents’ role during their adolescent’s pain journey, and the knowledge they gained; and (b) “Adapting and readjusting expectations,” capturing parents’ journeys and difficulties in learning to adjust to their adolescent’s pain experience. Themes are presented with relevant anonymized quotations to exemplify our interpretation of the data.

### Parental Perceptions of Being at the Heart of Pain Management

Parents perceived themselves as central to adolescents’ management of pain during their cancer journey. Pivotal to parents’ experiences was a need to understand adolescents’ pain, yet this was challenging in instances where adolescents did not clearly articulate the experience of pain, instead masking with stoic behavior, perhaps congruent with a more typical developmental trajectory whereby adolescents wish to reduce parental involvement. As one parent described; “It’s difficult to tell sometimes because she (participant’s daughter) doesn’t…give it away that she’s in pain…unless it’s really bad and then she will tell you” (P14, father of 16-year-old female; lymphoma). Such instances directly challenged parents’ ability to become expert in adolescent’s cancer-related pain, as an individual who is aware of their adolescent’s pain-related thoughts and perceptions. Perhaps a more successful aspect of parental aspirations for becoming experts in their adolescents’ pain related to supporting them to adopt a range of pain management strategies. This involved many parents liaising with professionals to find suitable techniques for managing adolescents’ cancer-related pain as exemplified by the following mother; “Getting help from all sorts of departments, with the anesthetists, rheumatologists, pain, and they’ve got the supportive and palliative team here … pulling on their expertise” (P1, mother of 16-year-old female; leukemia). Such information gathering provided a clear role for parents, enabling them to demonstrate expertise and credibility in helping adolescents to manage their pain, whilst recognizing the importance of healthcare professionals in being the primary source of their knowledge acquisition.

Challenges to parents’ perceived expertise arose when their adolescent had exhausted all pain management techniques available to them. This resulted in feelings of parental helplessness, challenging their perceived sense of responsibility to relieve adolescent’s pain; “It’s just so difficult to see your child hurt and not to be able to do anything about it” (P19, mother of 12-year-old male; brain tumor). Parents often felt frustrated when they had acquired knowledge about numerous pain management strategies, but had to acknowledge that adolescents were not necessary physically or mentally in a mindset to use such techniques. This contributed to parental helplessness, at the frustration of finding techniques that they thought would relieve adolescents of their pain, but ultimately adolescents were too unwell to use them; “She (participant’s daughter) found it very difficult to take on board… and employ those techniques when in the midst of it (experiencing high levels of pain)” (P12, mother of 15-year-old female; sarcoma). In these instances, parents felt compelled to adopt the role of “expert” as their adolescent seemed unable to assimilate and use this knowledge. Having devoted substantial time to developing their understanding of alternative pain management techniques to medication, it was often difficult for parents to comprehend that the knowledge they had acquired was insufficient in reducing adolescents’ pain.

In instances where parental suggestions of pain management strategies were deemed insufficient for managing adolescents’ pain, parents often resorted to physical touch to offer comfort in the presence of pain. Although such strategies were often seen as ineffective at alleviating pain, parents felt this was the only way they could offer support to adolescents, having tried all other options to manage the pain; “She (participant’s daughter) was in so much pain sometimes that all I could do was sort of hold her head because the rest of her body was hurting” (P2, mother of 17-year-old female; sarcoma). Fulfilling an important role of comforting their adolescent through the medium of touch supported the idea of parents adopting an expert role in their adolescent’s care, in knowing the ways in which they could still support adolescents after having exhausted all other strategies to alleviate the pain. Parents were uniquely placed in being able to offer such support, with one parent recognizing that although they had sought professional support to help relieve her son’s pain, this was not necessarily found to be beneficial to the adolescent; “Every hour we (participant and her husband) were sort of massaging his (participant’s son) limbs to try and help him out, um and I even booked him in for one of the (professional) massages…and he just said it (professional massage) was a bit weird really mum” (P19, mother of 12-year-old male; brain tumor). As evidenced in this quotation, this affords parents a unique role in pain management due to their knowledge around their adolescent’s needs and parental relationship with them.

An important aspect of parental mastery involved self-awareness of their need to step back, allowing adolescents themselves to manage their cancer-related pain to enable optimal pain relief. Parents understood the importance of recognizing adolescents as experts in their own pain and consequently, best able to identify the nature and function of their pain; *“*She (participant’s daughter) said she had pain in her leg, and I said, ‘Is it pre-diagnosis pain?’ and she went, ‘No, it’s this type’” (P5, mother of 18-year-old female; sarcoma). This communication was crucial to know how pain should be treated and the attention it required. Although parents were willing to enable adolescents to take control and remain autonomous in the identification and management of their pain, it was apparent that parents were ultimately adolescents’ biggest advocates and remained confident in their expertise in this role. An important aspect of parents’ expertise involved them judging in which instances it was appropriate for them to take control liaising with the healthcare professionals. Parents sometimes communicated with healthcare professionals on adolescents’ behalf, whilst also encouraging the adolescent’s own autonomy in this respect; *“*If they (doctors) say, ‘Oh well we’re back in half an hour’ and they don’t come back for two hours, go find the doctor… don’t give up…just be very very tenacious” (P1, mother of 16-year-old female; leukemia).

### Adapting and Readjusting Expectations

Parents described the challenges they faced when trying to adjust to a different life following their adolescent’s cancer diagnosis. They recognized how they had to change the ways they supported adolescents, and manage the resulting emotional difficulties. Evident throughout parental narratives was a sense of the continuing impact of cancer-related pain on parents and adolescents and the difficulties they faced in adapting their lives. Witnessing the far-reaching and deleterious impact of cancer-related pain on numerous aspects of adolescents’ lives was distressing for parents; “It interrupts her (participant’s daughter), it affects her appetite. It affects her mental wellbeing day-to-day or hour-to-hour. It is quite debilitating which is such a shame” (P1, mother of 16-year-old female; leukemia). Parents found the complete lifestyle change and loss of activities that adolescents experienced due to their pain difficult to witness, longing for adolescents to regain a sense of normality despite the pain. In addition to recognizing his own distress at seeing his daughter struggling, one father perceived his daughters’ experience of pain symptoms to be a trade-off, where she would only be able to cope with the pain if she could resume her regular teenage activities; “School was everything for her (participant’s daughter), it’s school friends, relationships, yes the biggest thing out of all of it, I’m sure she’ll…live with the pain and the fatigue as long as she can have her school life back” (P16, father of 14-year-old female; sarcoma).

Throughout adolescents’ pain journey, parents reported feeling frustrated at adolescents’ reluctance to ask professionals for support with their pain. Similar to parenting an adolescent without cancer, parents were constantly learning when it was appropriate for them to intervene and encourage adolescents to seek support. Where parents could be flexible with this, they noticed it usually benefited the adolescent and reduced their pain; *“*He (participant’s son) would sometimes say to me ‘Oh they’re really busy’ and I said, ‘But you’re still one of their patients it doesn’t matter that they’re busy if you need what you need then we need to press the button.’” (P8, mother of 16-year-old male; brain and germ cell tumors). Interestingly, although parents voiced frustrations that adolescents felt burdensome, parents felt unable to voice their own concerns, despite acknowledging that their perceived responsibility to help adolescents with their pain could be extremely stressful and distressing.

Parents were motivated to ensure that adolescents remained positive throughout the pain experience. This was highlighted by several parents reporting that their adolescent accepted that they would inevitably experience pain with a cancer diagnosis and as a consequence of procedures that were hoped to improve their health. Parents considered this acceptance to be beneficial in maintaining adolescents’ positivity; *“*He (participant’s son) seems to have a high tolerance of pain, and he said at the outset when he was diagnosed, he goes, ‘They can just do what they want with me. As long as it gets me better, I don’t mind’” (P3, father of 15-year-old male; leukemia). Due to the sudden changes to adolescents’ health and lifestyle as a consequence of the cancer diagnosis, parents held the perception that they had to immediately take control of their adolescent’s pain experience by providing them with increased parental support. However, over time, the realization and surprise that adolescents were able to power through and continue with their lives was welcomed by parents, and formed a central part of their readjustment of expectations; “She coped with it all in a way that I would never have imagined. I imagined having to pin her (participant’s daughter) down to the bed…I think she knew what was gonna happen, she intellectualised it all and went ‘OK’” (P7, mother of 18-year-old female; thyroid cancer). To manage the detrimental effects of pain on the adolescent and the family, many parents found it helpful to put the painful days in perspective, acknowledging that normality and daily functioning would resume once the pain subsided. One father explained; *“*We’d…say, ‘Look, it’s gonna be bad today, but this time next week your blood count should be up…What you’re experiencing now, it’s not gonna last for very long…we just gotta tick another day off’” (P3, father of 15-year-old male; leukemia). Ticking the days off acted as a coping mechanism for parents in knowing an improvement of adolescent’s pain was imminent, having recognized that the impact of adolescent’s physical symptoms and their distress was often carried on their shoulders.

Parents acknowledged substantial challenges in learning how to balance knowing where adolescents currently were developmentally, versus knowing what their adolescent’s needs really were. Throughout the cancer journey and as adolescents were getting older, parents were continually evaluating how best to support their adolescents, as one might when supporting any adolescent. Some parents perceived that their adolescent, whilst experiencing pain, reverted to requiring parenting techniques comparable to those used with a much younger child, developmentally conflicting with parenting an adolescent pre-cancer diagnosis. “I’d cuddle her (participant’s daughter) for ages, sometimes I’d get into bed with her and hold her…that need for physical comfort at all times stroking, holding, just being there” (P2, mother of 17-year-old female; sarcoma). Parents also recognized that they were at times hypervigilant toward possible signals from adolescents that might indicate that they were experiencing pain. This was akin to parental behavior associated with parenting a baby or toddler; “You become very aware, like at night_**…**_I wouldn’t sleep, every movement, every breath, you know I’d wake so easily, she’d (participant’s daughter) only have to make a noise and I was wide awake. It’s like going straight back to when they’re tiny_**…**_you’re in tune with everything they do” (P5, mother of 18-year-old female; sarcoma).

## Discussion

Having explored parents’ experiences of their adolescent’s cancer-related pain during and shortly after completing cancer treatment, our results highlighted how parents learned to support adolescents to manage their pain, and the emotional difficulties and developmental conflicts that parents experienced. Many parents reported that healthcare professionals were key to increasing theirs and their adolescents’ knowledge about pain management, including medication and alternative strategies. Importantly, several parents noted the necessity of ensuring adolescents continued with their lives as normally as possible despite the pain. Aiding this was an overwhelming sense of parents’ encouragement and advocacy during their adolescents’ cancer treatment. Despite this, parents voiced feelings of helplessness, stemming from the frustration of trying multiple pain management strategies without adolescents experiencing pain relief. Many parents also recognized their adolescent’s need for more help when they were experiencing cancer-related pain compared to typical adolescents, which required a shift in the support parents provided to them.

Although some research has identified that pain is often self-managed without input from healthcare professionals (Linder et al., 2017), participants in the present study reported that professionals were crucial in this learning process. However, the finding that parents frequently mentioned the importance of gaining mastery over family life and adjusting their lives around their adolescent’s pain, has been highlighted in previous cancer literature (Björk et al., 2005). In line with other studies, one of the main components of the perceived parental role was advocating for adolescents throughout treatment (Tutelman et al., 2019; Twycross et al., 2015). This was particularly the case in ensuring adolescents received the best treatment possible, driven perhaps by a sense of parental responsibility and fear, especially if the adolescent experienced complications during diagnosis or treatment (Twycross et al., 2015). However, parental helplessness was consistent with many studies involving parents of children and adolescents with cancer (Alberts et al., 2018; Rodriguez et al., 2012), where helplessness stemmed from frustrations of trying multiple pain management techniques without adolescents experiencing pain relief. Another important finding was the frequently mentioned difficulties parents experienced when adolescents were not vocalizing pain. This has been discussed in other adolescent cancer-related research, where adolescents often prefer to cope with pain alone to avoid causing parents additional stress or facing restrictions themselves if they voice the pain (Ameringer, 2010; Parker et al., 2020).

Adolescents experiencing cancer-related pain disrupt the typical developmental trajectory of increasing independence, instead becoming more reliant on their parents whilst they undergo treatment (Drew et al., 2019). Consistent with this, parents reported supporting adolescents considerably more than before diagnosis. Broader chronic pain research highlights the distress parents face engaging with non-normative teenage behaviors and caring responsibilities (Maciver et al., 2010); yet few cancer-related studies address this phenomenon. Although parents in the present study longed for adolescents to resume their pre-cancer “normal” lives, parents were happy to provide requisite physical support to their adolescent, more akin to parenting a younger child. In fact, many parents were comforted by the increasing closeness associated with supporting adolescents to manage cancer-related pain, indicating the easing of a sense of loss of this close relationship as adolescents had become more independent prior to their diagnosis. Consequently, parents reported challenges in disengaging from providing this more child-like support. This may also be due to the hypervigilance many parents of adolescents with cancer-related pain experience (Drew et al., 2019), associated with constant worrying and an inability to switch off (Rodriguez et al., 2012). Many take a “better safe than sorry” approach (Heathcote & Eccleston, 2017), overestimating the severity of pain symptoms. As parents did not allude to adolescents being overly concerned about their pain, this perhaps supports the view that parents are more likely to interpret pain as threatening (Tutelman et al., 2019). Similar to Woodgate and Degner (2003), the results of the present study revealed that many adolescents accepted pain as an inevitable symptom of cancer, and a fundamental component of receiving treatments. They specifically found that parents and adolescents adopted a “short-term pain, long-term gain” attitude toward pain; interestingly the present study found that parents seemed surprised at adolescents’ ability to accept pain as inevitable and power through the pain. This could have related to a surprise at adolescents’ maturity in how they coped throughout their cancer journeys, as opposed to parents themselves not accepting that pain would be inevitable.

### Study Strengths and Limitations

Investigating parental experiences was a notable strength. Having been neglected somewhat in the literature, the parental voice is shown to be essential in understanding adolescents’ experiences. Including participants up to a year post-treatment was a particular strength; this period is often neglected in pain studies, despite a pain prevalence of 5–59% in childhood cancer survivors (Alberts et al., 2018). The present findings would be strengthened by inclusion of adolescents’ perspectives. Despite the apparent congruence of parent–child narratives (Parker et al., 2020), adolescents may mask pain and try to manage symptoms without parental support (Ameringer, 2010), perhaps compromising the accuracy of parents’ accounts. The sample predominantly comprised mothers, meaning findings may not represent fathers’ experiences. Some studies have found disparities between the reporting of adolescents’ pain symptoms, where mothers rate pain of a greater severity and distress (Hedén et al., 2013). It is therefore important that a more equal gender balance is obtained in future research studies. Demographic data relating to race and ethnicity were not collected in the present study. Despite considering only adults, Kwok and Bhuvanakrishna (2014) revealed that Black individuals were twice as likely to report severe pain compared to White individuals. Furthermore, certain Eastern cultures normalize pain experiences, whereas individuals in Western cultures are more likely to seek help when they experience pain. The collection of such data in future studies is crucial to know where differences in perceptions of cancer-related pain lie between cultural and ethnic groups. A further possible limitation was the use of purposive sampling in recruitment. Parents approached may have had more trusting relationships with clinicians (Coyne et al., 2016), thus more positive experiences.

### Implications for Research and Clinical Practice

The findings highlight the necessity of a multidisciplinary approach to managing pain, involving adolescents, healthcare professionals, and parents where appropriate and necessary (Uhl et al., 2020). This approach encourages the best possible support, integrating those who know the adolescent best with individuals offering specialized knowledge. Considering the age range in the present study, it is important to acknowledge that adolescents’ needs may vary across the developmental period. Healthcare professionals must also consider that parental involvement may lead to an increase in adolescents’ anxiety, and consequently higher levels of pain (Alberts et al., 2018). As such, it is advisable to offer a review at regular intervals through treatment, with adolescents and their families, supporting the update of independence versus parental involvement at each stage of development, taking into account adolescents’ needs and preferences. Regardless, discussions and information must be shared with adolescents (and parents where helpful) in developmentally appropriate formats, to aid adolescent’s transition to greater control over their care. Research studies piloting transition pathway programs within other long-term health conditions, such as Juvenile Idiopathic Arthritis, have identified benefits of enabling adolescents to discuss symptom management and their care with a transition co-ordinator (Hilderson et al., 2016). Promoting autonomy by adolescents discussing concerns with professionals gave parents confidence to hand responsibility to adolescents, reducing their involvement over time. Recognizing the understandable challenges and feelings of powerlessness parents may face (Hilderson et al., 2016), educating and supporting them in the lead-up to the transition can help parents feel more comfortable stepping back to allow space for independence (Bashore & Bender, 2016).

To facilitate this, future studies should continue to explore different cancers and identify the support needed by adolescents and parents during and after treatment. This will help to better equip healthcare services to provide the right care at the right times, in liaison with other sectors (e.g., schools), to ensure families feel well supported and adolescents are able to access all areas of life. As parents can be reluctant to seek help for themselves, it is important that healthcare professionals routinely check in with how they are feeling and offer support options where necessary, for example, when faced with a sense of responsibility, frustration, or helplessness. If parents feel well supported, they are likely to feel more confident in the caring role, reducing the unplanned, anxiety-led contact with healthcare services when they are unsure how to help adolescents at home. Improving our understanding of how parents and adolescents can be supported during the cancer journey will go some way to improving the provision of resources, the lived experience of families through treatment, and ultimately lead to better long-term physical and psychological outcomes.

## Data Availability

The data underlying this article cannot be shared publicly for the privacy of individuals that participated in the study.
